# Analysis of muscle tissue *in vivo* using fiber-optic autofluorescence and diffuse reflectance spectroscopy

**DOI:** 10.1117/1.JBO.26.12.125001

**Published:** 2021-12-22

**Authors:** Christopher J. Davey, Emily R. Vasiljevski, Alexandra K. O’Donohue, Simon C. Fleming, Aaron Schindeler

**Affiliations:** a University of Sydney, Institute of Photonics and Optical Science, School of Physics, Sydney, New South Wales, Australia; b The Children’s Hospital at Westmead, Bioengineering and Molecular Medicine Laboratory, Westmead, New South Wales, Australia; c University of Sydney, Sydney Medical School, Discipline of Child and Adolescent Health, Sydney, New South Wales, Australia

**Keywords:** autofluorescence spectroscope, diffuse reflectance spectroscopy, muscle, myopathy, muscular dystrophy, *in vivo* spectroscopy, *in vivo* fiber-optics

## Abstract

**Significance:** Current methods for analyzing pathological muscle tissue are time consuming and rarely quantitative, and they involve invasive biopsies. Faster and less invasive diagnosis of muscle disease may be achievable using marker-free *in vivo* optical sensing methods.

**Aim:** It was speculated that changes in the biochemical composition and structure of muscle associated with pathology could be measured quantitatively using visible wavelength optical spectroscopy techniques enabling automated classification.

**Approach:** A fiber-optic autofluorescence (AF) and diffuse reflectance (DR) spectroscopy device was manufactured. The device and data processing techniques based on principal component analysis were validated using *in situ* measurements on healthy skeletal and cardiac muscle. These methods were then applied to two mouse models of genetic muscle disease: a type 1 neurofibromatosis (NF1) limb-mesenchyme knockout ($Nf{1_{Prx1}}^{-/-}$) and a muscular dystrophy mouse (*mdx*).

**Results:** Healthy skeletal and cardiac muscle specimens were separable using AF and DR with receiver operator curve areas (ROC-AUC) of >0.79. AF and DR analyses showed optically separable changes in $Nf{1_{Prx1}}^{-/-}$ quadriceps muscle (ROC-AUC >0.97) with no differences detected in the heart (ROC-AUC <0.67), which does not undergo gene deletion in this model. Changes in AF spectra in *mdx* muscle were seen between the 3 week and 10 week time points (ROC-AUC = 0.96) and were not seen in the wild-type controls (ROC-AUC = 0.58).

**Conclusion:** These findings support the utility of *in vivo* fiber-optic AF and DR spectroscopy for the assessment of muscle tissue. This report highlights that there is considerable scope to develop this marker-free optical technology for preclinical muscle research and for diagnostic assessment of clinical myopathies and dystrophies.

## 1 Introduction

Optical technologies capable of real-time quantitative analysis of tissue structure and biochemical composition have the potential to revolutionize medical diagnostics. Histology is the gold standard for clinical diagnostics, but it can often involve painfully invasive biopsies, long turn-around times, and qualitative decision making. Established noninvasive imaging technologies, such as computed tomography, magnetic resonance imaging, and ultrasound, offer faster, reliable, and easily interpreted anatomical data with some compositional information. However, these modalities suffer from a variety of limitations including long acquisition times, ionizing radiation exposure, poor contrast between soft tissue types, the need for use of exogenous contrast agents, and large and expensive hardware.^1^^–^^3^ Alongside these methods, a range of optical techniques that utilize custom-engineered exogenous fluorescent markers of disease have emerged; these include small molecule organic fluorophores,^4^ quantum dots,^5^ carbon nanotubes,^6^ and various other nanomaterials.^7^^,^^8^ However, considerable scope remains for developing minimally invasive spectroscopic devices [e.g., based on autofluorescence (AF) or diffuse reflectance (DR)] that can be used in the absence of specific probes or dyes.

AF spectroscopy involves exposure of tissues to monochromatic ultraviolet (UV) or visible light and the collection of the Stokes-shifted spontaneous emission spectrum that originates from endogenous fluorophores. Naturally fluorescent biological molecules include a variety of amino acids, structural proteins, enzymes, coenzymes, vitamins, lipids, and porphyrins, each of which possesses distinctive excitation/emission spectra.^9^^,^^10^ Their distributions vary between tissue types, and their individual fluorescence properties can be affected by their molecular environment,^11^ pH,^12^ and temperature,^13^ making AF spectroscopy a powerful diagnostic tool. This may be complemented by DR spectroscopy, which uses broad-spectrum illumination and detection of diffusely reflected photons over the same wavelength range. DR spectroscopy is sensitive to absorption by endogenous chromophores, including hemoglobin and myoglobin,^14^ as well as light scattering from micron-scale structures, such as cell membranes, nuclei, and mitochondria.^15^

In this study, we aimed to investigate the utility of AF/DR spectroscopy in the context of muscle disease. Myopathies represent a major disease burden in which early diagnosis can be immensely helpful in terms of future clinical management. Myopathies have numerous characteristic histological features that are currently difficult to detect *in situ* and can only be reliably identified using invasive biopsies. Evidence of a congenital myopathy can be seen in the presence of nemaline rods,^16^ fiber-type disproportion,^17^ intramyocellular lipid,^18^ Z-line streaming, and sarcomeric disruption.^19^ Muscular dystrophies often involve adipocyte or fibrotic invasion, the presence of which can correlate with progressive muscle weakness.^20^ Both quantitative and qualitative histology techniques exist;^20^^,^^21^ however, both are time consuming, and biopsy collection is associated with tissue morbidity, pain, infection risk, long turn-around times, and error-prone logistics.^22^

There is reason to expect that a spectroscopic approach will be effective in characterizing muscle tissue. In skeletal muscle, unique AF signatures of individual myofiber types have been demonstrated, enabling classification of whole muscle types with unique heterogeneous myofiber compositions.^23^^–^^25^ Differences in their metabolic biochemistry are thought to alter the fluorescence properties of mitochondrial fluorophores including nicotinamide adenine dinucleotide (NADH) and flavin adenine dinucleotide (FAD), as well as some essential amino acids including tyrosine and tryptophan. This technique may also be applicable to detecting changes in muscle composition with disease and aging. Nakae et al.^26^ demonstrated the accumulation of fluorescent lipofuscin granules from oxidative breakdown of cellular macromolecules in human and murine dystrophic and aged myofibers detectable with visible wavelength AF microscopy. Further, the sensitivity of AF spectroscopy to muscle fat and collagenous tissues^25^^,^^27^ may be relevant to the diagnosis of muscle diseases for which intracellular adiposity and fibrosis are phenotypes.^18^^,^^28^

A current barrier to widespread use of this technique is a lack of economical, accessible, and easy-to-use platforms that integrate AF/DR spectroscopy with relevant and easily interpretable spectral analyses. In principle, such an approach is feasible and supported by a range of studies characterizing tissue AF in the gut, arteries, and myocardium^29^^–^^31^ and in tumors.^32^^–^^35^ AF spectroscopy has also been employed in conjunction with DR spectroscopy in a range of settings.^36^^–^^39^ Athough there is currently a lack of information on the use of AF/DR spectroscopy within the specific context of diseased muscle tissue, AF/DR probes have been developed to identify commercial meat cut quality based on intermuscular collagen and adipose content.^14^^,^^25^^,^^27^

In this study, we describe a fiber-optic AF/DR system and speculate that both modalities may be capable of real-time marker-free classification of healthy and diseased skeletal muscle tissues. The system was trialed using several established preclinical models of muscle pathology. The *mdx* mice are a widely used model of muscular dystrophy that feature a mutation in the murine *dystrophin* gene.^40^ Like in the clinical scenario, *mdx* mice exhibit a refractory period in which they are asymptomatic before developing muscle weakness. The $Nf{1_{Prx1}}^{-/-}$ mouse line is a limb-specific knockout mouse line in which the NF1 gene is deleted in mesenchymal tissues including muscle.^41^ NF1 is critical for muscle development and function, and NF1-deficient muscle features intramyocellular lipid accumulation and fibrosis.^18^^,^^28^ Data preprocessing and classification techniques were first optimized with the goal of classifying optical measurements from different muscles groups in healthy wild-type mice before these techniques were applied to the classification of muscle tissues from mouse models of muscle pathology. These results were compared with standard histological techniques for muscle assessment.

## 2 Materials and Methods

### 2.1 Optical System

**Fig. 1:**
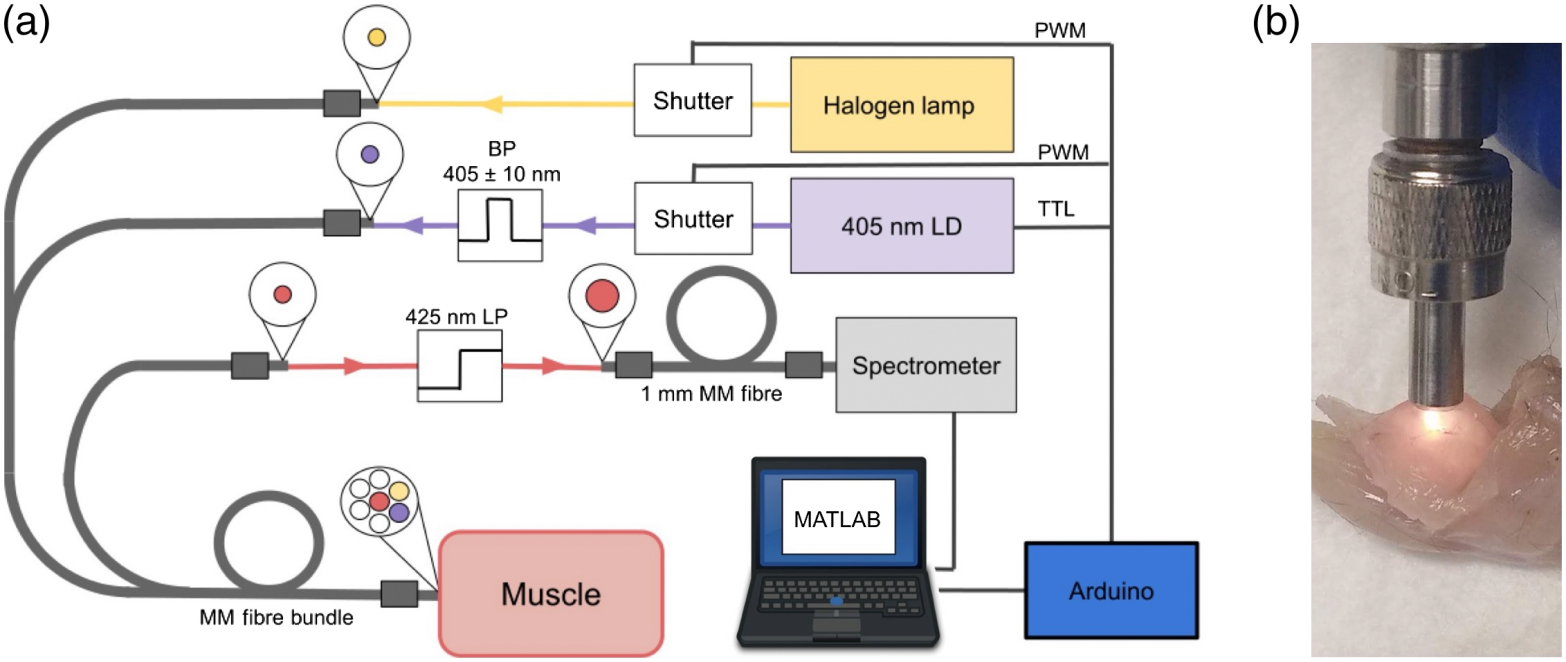
(a) Schematic of the fiber-optic AF/DR system. 405-nm laser (violet) and halogen lamp (yellow) light sources were coupled into proximal ends of two multimode (MM) optical fibers via servo-mechanical shutters and a bandpass (BP) filter to further narrow the AF excitation wavelength range. Tissue DR and AF light was collected via a central fiber at the probe’s distal end (red) and passed through a 425-nm long-pass (LP) filter before detection at the spectrometer. Additional fibers within the fiber-optic probe (white) were unused. (b) The fiber-optic probe head was positioned directly onto the muscle surface during optical measurements, here showing a DR measurement on the murine gastrocnemius.

A custom AF/DR system was built using commercially available components. A 200-mW 405-nm laser diode (LD) was used for AF measurements, coupled via a servo-actuated shutter, bandpass filter (FB405-10, Thorlabs), and microscope lens into one of seven multimode optical fibers (200-μm core diameter and 0.39 NA) within a fan-out style bundle (BF72HS01, Thorlabs) terminating distally in a six-around-one configuration [Fig. 1(a)]. The shutter allowed the laser power to ramp-up and stabilize before each measurement, while illumination power, set to 20 mW, was controlled by adjusting the coupling between the fiber and LD. Tissue AF emission was collected using the bundle’s central fiber and long pass filtered at 425 nm (DMLP425, Thorlabs) to remove the illumination wavelength before detection by a USB spectrometer with a wavelength range of 345 to 1041 nm (USB 4000, Ocean Optics).

A stabilized tungsten–halogen light source (SLS201L/M, Thorlabs) was used for DR measurements, coupled via a servo-actuated shutter and microscope lens into a fiber adjacent to the 405-nm LD [Fig. 1(a)]. Diffusely reflected light was collected using the same central fiber as for AF measurement and returned to the same spectrometer via the same optical path. The four remaining fibers within the bundle were unused. System control and data collection were performed on a PC laptop using MATLAB (Mathworks) via direct USB connection to the filter wheel and spectrometer. An Arduino was used for transistor–transistor logic and pulse-width modulation signals for control of the LD and shutter servos, respectively.

### 2.2 Animal Ethics and Husbandry

C57BL/6 mice (B6) and $Nf{1_{Prx1}}^{-/-}$ mice (NF1) were bred in-house at the Transgenic Facility at The Children’s Hospital at Westmead. B6 mice were originally sourced from the Animal Resources Centre (ARC, Perth), and the NF1 mice bred from the *Prx1-Cre* transgenic mice^42^ and the $Nf1^{flox/flox}$ mice.^43^ $Nf{1_{Prx1}}^{-/-}$ were bred on a B6 background. The *mdx* mice (B10*mdx*) were sourced from Australian Bioresources (ABR, Moss Vale). As these mice are on a C57BL/10 background, wild-type mice (B10WT) of this strain were also sourced from ABR. These studies were approved by the local Animal Ethics Committee under protocol K319.

### 2.3 *In-Vivo* Measurements

Measurements were made across six groups of mice defined by their genetic strain and age: B6 at 10 weeks of age, NF1 at 10 weeks, B10WT at 3 and 10 weeks (B10WT-3 weeks/10 weeks), and B10*mdx* at 3 and 10 weeks (B10*mdx*-3 weeks/10 weeks). Each group comprised five mice, from which multiple measurements were taken across different muscle groups and the left and right sides. Mice were euthanized immediately prior to measurement by ${\mathrm{CO}}_{2}$ asphyxiation. The quadriceps (Quad), tibialis anterior (TA), and gastrocnemius (Gastr) of both hind limbs were exposed using surgical scissors, removing outer connective tissues including the superficial fascia. The distal tip of the optical fiber bundle was positioned in the center of and in direct contact with each muscle [Fig. 1(b)], and AF/DR measurements, including background measurements without illumination for calibration, were made successively. Measurements were made on each muscle in triplicate, lifting and repositioning the probe on the same muscle for each repetition. Following a sternotomy, the heart was exposed for a further set of measurements on the lateral edge of the left and right ventricles.

### 2.4 Histology

Muscle tissues were harvested from all mice following optical measurement. These tissues were surface coated in Tissue-Tek^®^ O.C.T. Compound (Sakura Finetek), frozen in liquid nitrogen supercooled isopentane (2-methyl butane), and stored at −80°C. 8-μm sections were cut on a Leica CM1950 Clinical Cryostat, captured on Superfrost™ Plus Microscope Slides (Fisher Scientific), and stored at 4°C prior to staining. H&E and Oil Red O staining were performed as previously published.^18^

### 2.5 Optical Data Preprocessing

All data processing and analysis were performed in MATLAB (Mathworks). All spectra were calibrated by subtraction of corresponding background spectra to remove the influence of ambient room light. DR spectra were further calibrated to account for the broadband source spectrum as measured from a polytetrafluoroethylene (PTFE) block, such that $X=(X_{R}-B)/W$, where X is the calibrated spectrum, $X_{R}$ is the measured tissue spectrum, B is the background spectrum taken from the tissue without illumination, and W is the PTFE spectrum. PTFE is known to have a flat reflectance spectrum across the visible and near-infrared region and is hence highly suitable as a white reference material.^44^ Calibrated spectra with 3648-data points corresponding to each detection wavelength of the spectrometer were then smoothed using a moving average filter with a window size of 10 nm. AF and DR spectra were then windowed between 435 to 900 nm and 450 to 900 nm, respectively, to remove artifacts and noise at the spectral extremes. An analysis based on spectral shape rather than original calibrated intensities was speculated to be less affected by probe contact quality and light source intensity fluctuations. Calibrated spectral data were normalized to the wavelength of maximum intensity such that $X_{N}=X/\max(X)$, where $X_{N}$ is the normalized spectrum. Spectra were then grouped into matrices according to their animal- or muscle-group class labels, enabling the comparison of any combination of genotype, muscle group, and age as required for subsequent analyses.

### 2.6 Optical Feature Extraction

Dimensionality reduction of spectral matrices was achieved using principal component analysis (PCA)^45^ similar to other studies^46^^,^^47^ as detailed in Supplemental Methods 1. This method removes noise from the dataset and allows the visualization of important spectral regions that account for variance in the original dataset. Manual feature extraction methods, e.g., calculating intensities at specific wavelengths of interest, ratios of intensities at different wavelengths,^32^^,^^48^ integrated spectral intensity,^32^ or analyzing derivatives,^49^ were avoided to prevent significant loss of variance and potential nonorthogonality between features. For each dataset, spectral features known as principal components (PCs) were extracted, with each being a linear combination of the detection wavelengths. Low variance-explaining PCs were discarded to leave the first three PCs onto which each original observation was projected to give a score matrix with three variables per observation. A preliminary investigation indicated that the first three PCs explained a sufficient percentage of total variance (>97%) when applied to normalized AF and DR measurements across each muscle type in the B6 mouse group (Supplemental Methods 2). Normalization of calibrated spectral data provided improved separation of measurement scores enabling better classification accuracy, as has been demonstrated in other studies.^46^

### 2.7 Classification Model

A quadratic discriminant classifier (QDC)^50^ was then used for classification of the PC score matrices. For each classification task, a QDC was trained using fivefold cross validation as described in Supplemental Methods 1. The area under the curve of the model’s receiver operator curve (ROC-AUC) was used as a general measure of prediction accuracy.^51^

## 3 Results

### 3.1 AF and DR Spectra Enable Separation of Different Muscle Types in Wild-Type Mice

**Fig. 2:**
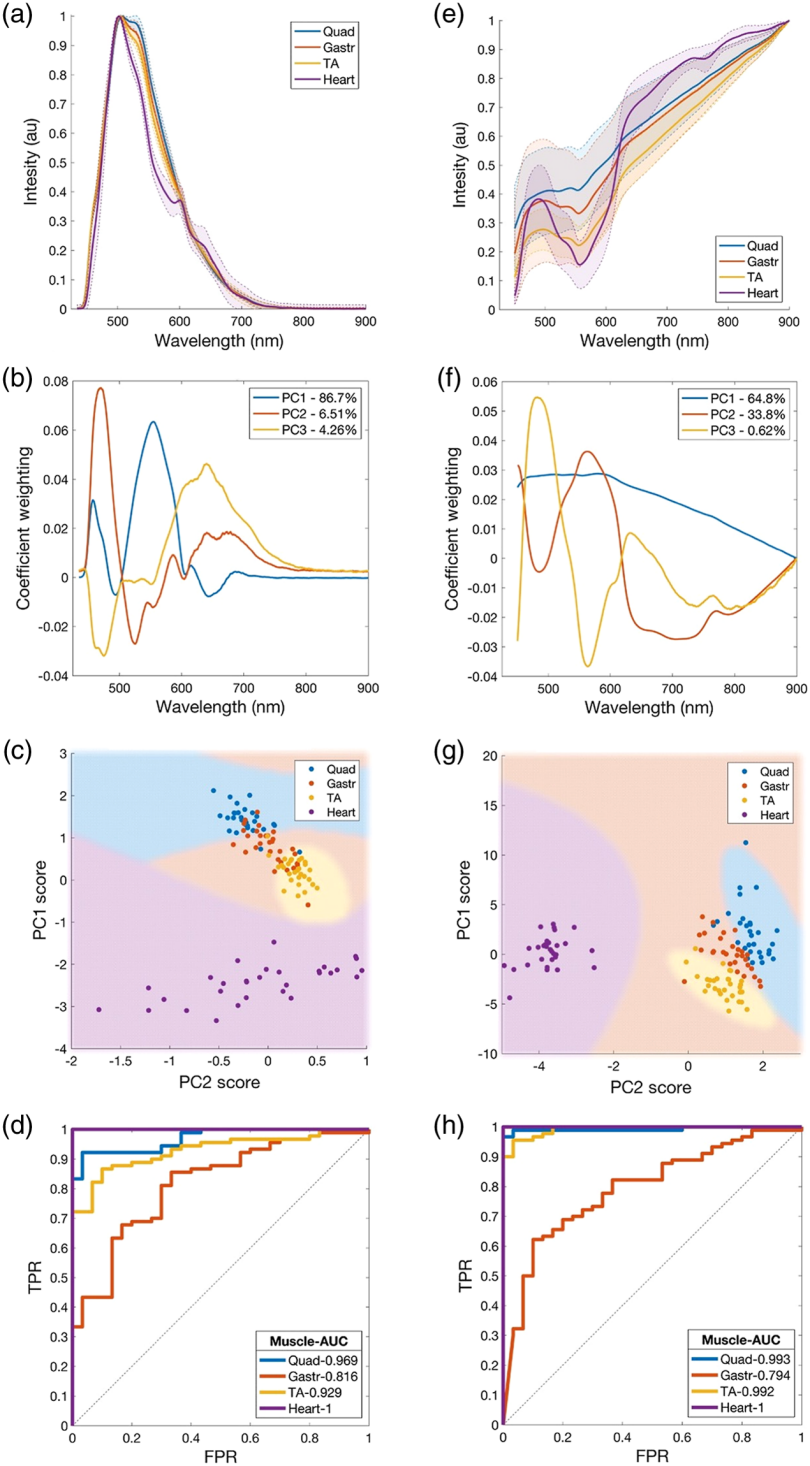
(a)–(d) AF and (e)–(h) DR spectroscopy enabled classification of muscle types (Quad, Gastr, TA, and heart) in healthy B6 mice using PCA. (a), (e) Average normalized spectra (solid lines) show spectral differences between quadriceps, gastrocnemius, tibialis anterior, and cardiac muscle (95% CIs shown by dotted lines and shaded areas). (b), (f) Coefficient weightings of each principal component describe sources of variance across all wavelengths, including each PC’s total percentage explained variance (see legend). (c), (g) PC1 and PC2 scores show clustering of muscle types, which enabled their classification using decision boundaries (shaded regions) designed by a QDC. (d), (h) Classification ROC curves provide a quantitative measure of muscle type separability, where an AUC of 1 describes a perfect classifier and 0.5 a weakest classifier (see legend for muscle-specific ROC-AUCs).

It was hypothesized that different muscle groups that contain different fiber types and cytoskeletal proteins would produce different AF and DR spectra. Measurements were made from several murine hind limb muscles (gastrocnemius, quadriceps, and tibialis anterior) as well as from cardiac muscle from the same animals. Statistical analysis of normalized spectra was applied to generate 95% confidence intervals (CIs), which indicated the separability of each muscle group at each detection wavelength. For AF measurements, overlapping CIs were observed across all wavelengths for skeletal muscle groups, whereas cardiac muscle showed significantly different spectral shape to skeletal muscles across the 500 to 600 nm range [Fig. 2(a)]. For DR measurements, overlapping CIs were observed across all wavelengths for all skeletal muscle groups, whereas cardiac muscle was separable from only quadriceps and gastrocnemius groups at ∼560  nm, and from tibialis anterior only between 630 and 895 nm [Fig. 2(e)].

To enhance muscle group separability and resulting classification accuracy, spectra were dimensionally reduced using PCA. The coefficient weightings for the first three components of AF and DR spectra are shown in Figs. 2(b) and 2(f), respectively, whereby large (non-zero) weightings indicate sources of variance between observations. For AF spectra, prominent weightings were observed at 475, 550, and 650 nm and for DR spectra at 490 and 570 nm.

These spectral differences caused muscle-type specific clustering of AF and DR PC scores as shown in PC1 versus PC2 score space [Figs. 2(c) and 2(g)]. Skeletal muscles formed mildly overlapping clusters with intercluster variance across both PC1 and PC2, whereas cardiac muscle formed a separate cluster best distinguished from skeletal muscles through PC1 in AF measurements, and PC2 in DR measurements. PC3 variance is not visualized in these figures but was also used for the purpose of classification.

The classification of muscle types was performed using a QDC classifier with fivefold cross validation. The classifier’s performance for each muscle type was quantified using the area under the curve (AUC) of its ROC curve [Figs. 2(d) and 2(h)], while its decision boundaries were observed in the PC scores plots [Figs. 2(c) and 2(g)]. The cardiac muscle was well separable from all skeletal muscles in both AF and DR analyses (AUC=1 and AUC=1 for AF and DR, respectively), whereas skeletal muscles (Quad, Gastr, and TA) were less separable with AUCs ranging between 0.79 and 0.99.

### 3.2 AF/DR Spectra Enable Separation of Healthy Muscle and Muscle Containing Intramyocellular Lipid

**Fig. 3:**
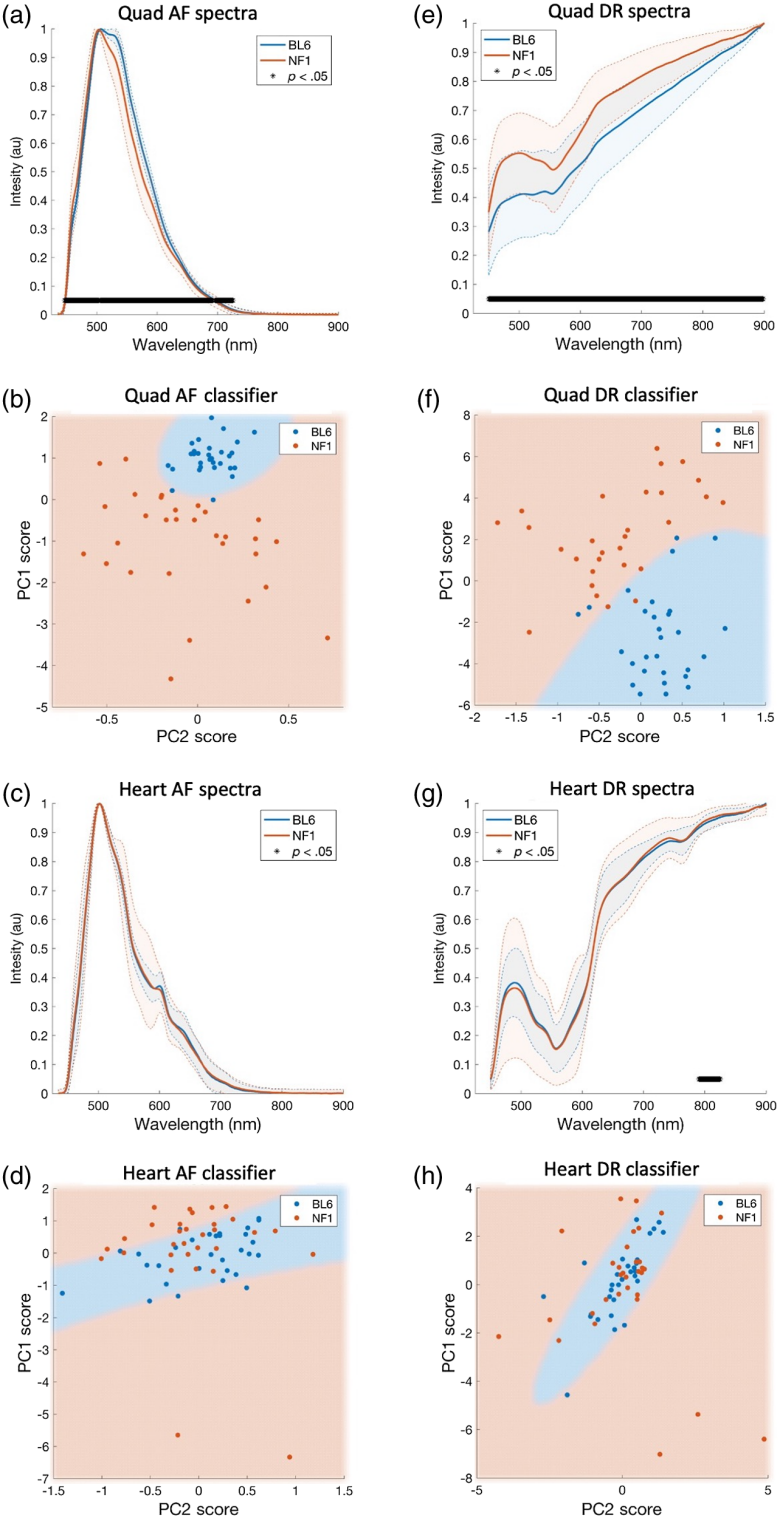
(a)–(d) AF and (e)–(h) DR spectroscopy of quadriceps muscle enables classification of NF1 mice from B6 controls (both 10 weeks) using PCA scores; however, when applied to cardiac muscle, the same analysis yields no separability. Normalized AF spectra of (a) quadriceps and (c) cardiac muscle in B6 and NF1 mice with [(b), (d)] corresponding PC score and QDC decision boundary plots. Normalized DR spectra of (e) quadriceps and (g) cardiac muscle in B6 and NF1 mice with [(f), (h)] corresponding PC score and QDC decision boundary plots.

It was next tested whether AF/DR spectra could separate healthy versus pathological muscle tissue in murine models of muscle disease. NF1 has shown features of a metabolic myopathy,^52^ and histological staining of quadriceps muscle in $Nf{1_{Prx1}}^{-/-}$ revealed an accumulation of intramyocellular lipid.^18^^,^^28^ Cardiac tissue was included as a negative control as the *Prx1* promoter does not allow for expression in the heart. Analysis was performed on B6 and NF1 mice (10 weeks) using AF and DR measurements (Fig. 3).

**Fig. 4:**
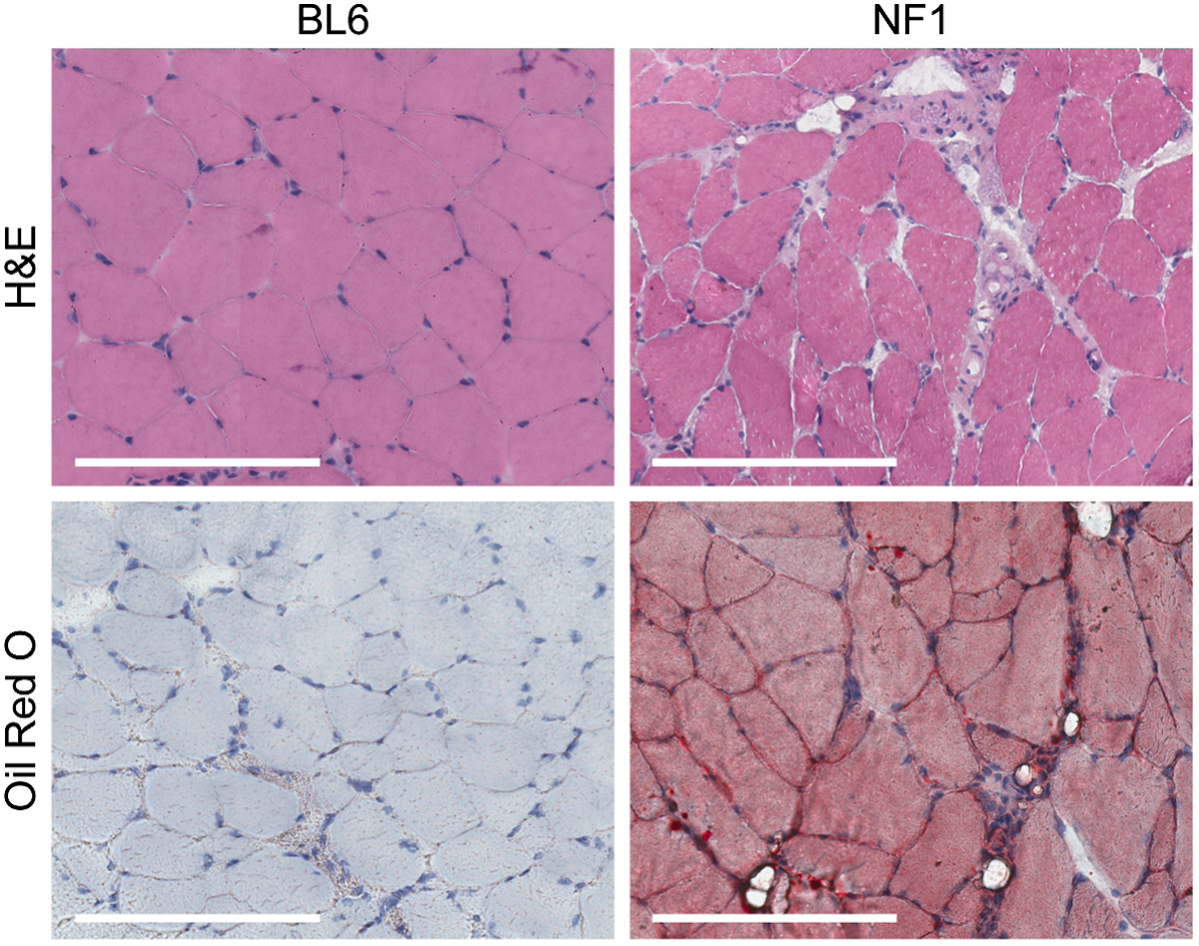
Histopathology of quadriceps muscle from 10-week-old NF1 mice differs from that observed in B6 mice. H&E staining shows endomysial fibrosis, and Oil Red O staining illustrates the intramyocellular lipid seen in NF1 mice. Scale bars indicate 200  μm.

Normalized AF spectra from B6 and NF1 quadricep muscles showed significantly different means (p<0.05 between ∼450 and 720 nm) [Fig. 3(a)]. NF1 DR spectra showed a more pronounced absorption feature between 500 and 600 nm, whereas both mouse groups showed different means across the entire wavelength range (p<0.05). Both AF and DR demonstrated strong quadriceps separability as indicated by distinct PC score clustering [Figs. 3(b) and, 3(f)] and QDC classifier AUC’s of 0.99 and 0.97, respectively. B6 and NF1 cardiac muscle showed similar spectral shapes and the same mean normalized intensity across almost all wavelengths for both AF and DR (p>0.05) [Figs. 3(c) and 3(g)]. This similarity resulted in overlapping PC score clusters [Figs. 3(d) and 3(h)] and poor QDC classification performance with ROC-AUCs of 0.67 and 0.63 for AF and DR, respectively. *NF1* muscle histology (Fig. 4) showed endomysial fibrosis and intramyocellular lipid by H&E and Oil Red O staining, respectively, consistent with prior findings.^18^

### 3.3 AF/DR Spectra Provide Separation of In Predystrophic and Dystrophic Muscle

A similar analysis was performed on B10*mdx* mouse groups at two different time points using the B10WT groups as controls. At 3 weeks, *mdx* mice are known to be asymptomatic and show no major histological features of dystrophy. By 10 weeks, *mdx* mice show centralized nuclei and fibrosis characteristic of dystrophic muscle. It was queried whether any fundamental differences in the AF/DR spectra would be apparent in *mdx* mice before histological features were apparent, as well as at later stages of muscle degeneration/regeneration.

**Fig. 5:**
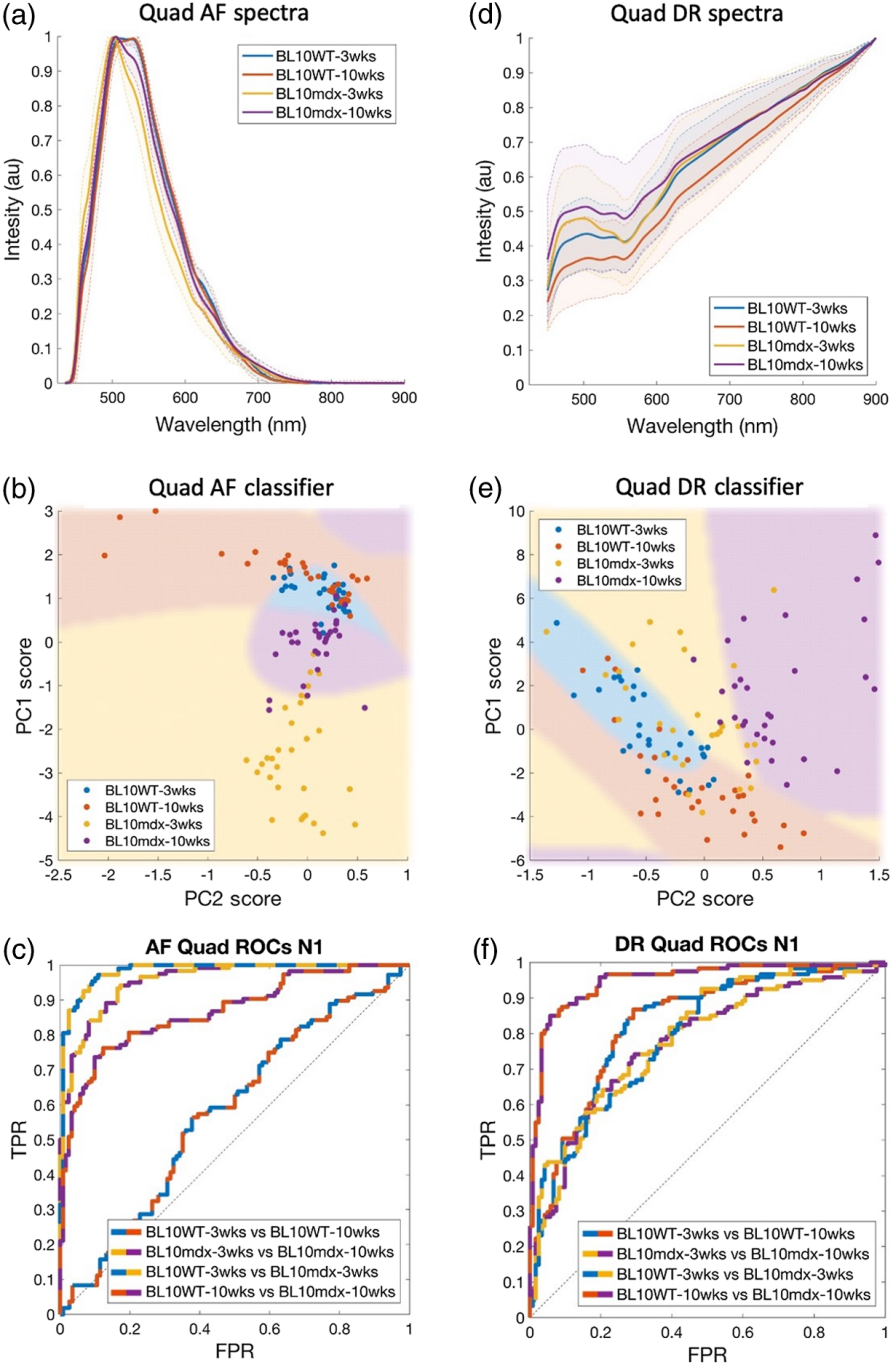
(a)–(c) AF and (d)–(f) DR spectroscopy on quadriceps muscle enabled classification of *mdx* mice from B10 controls using PCA at both 3 week and 10 week time points. Normalized AF and DR spectra (top row) indicate differences in optical properties between these groups. These differences are highlighted by clustering in PCA space (middle row) as well as in their classification ROC curves (bottom row).

AF and DR measurements were made on the quadriceps of B10WT and B10*mdx* mice at 3 and 10 weeks [Figs. 5(a) and 5(d)]. The PCA-QDC method was again used to test separability of different pairs of these groups. First, B10WT groups at 3 and 10 weeks were compared. Both groups showed similar AF spectral features [Fig. 5(a)] with overlapping PC Score clusters [Fig. 5(b)] making them indistinguishable to the QDC [Fig. 5(c)] with an ROC AUC=0.58 (Table 1). DR measurements provided better separability, AUC=0.83. In contrast, B10*mdx* at 3 and 10 weeks showed differences in AF spectral features resulting in strong classifier performance, AUC=0.96, whereas DR spectra were only modestly separable, AUC=0.77. Further comparisons were made between control B10WT and B10*mdx* mice, both at 3 weeks, with strong AF separability, AUC=0.98 and with more modest DR separability, AUC=0.73. Finally, B10WT and B10*mdx* mice were compared at 10 weeks, with similar results to that of 3 weeks, with stronger separability using AF, AUC=0.87 and modest separability using DR, AUC=0.77.

**Table 1:** DC ROC-AUC metrics for pairwise classification of B10WT and B10*mdx* mouse groups at 3 and 10 weeks. AUCs were used as a measure of class separability, where AUC>0.9 = strong separability, 0.9>AUC>0.7 = modest separability, and AUC<0.7 = poor separability.

Compared groups	QDC ROC-AUC	
	AF	DR
B10WT 3 weeks versus 10 weeks	0.580	0.826
B10*mdx* 3 weeks versus 10 weeks	0.956	0.774
B10WT versus B10*mdx* at 3 weeks	0.982	0.793
B10WT versus B10*mdx* at 10 weeks	0.868	0.774

**Fig. 6:**
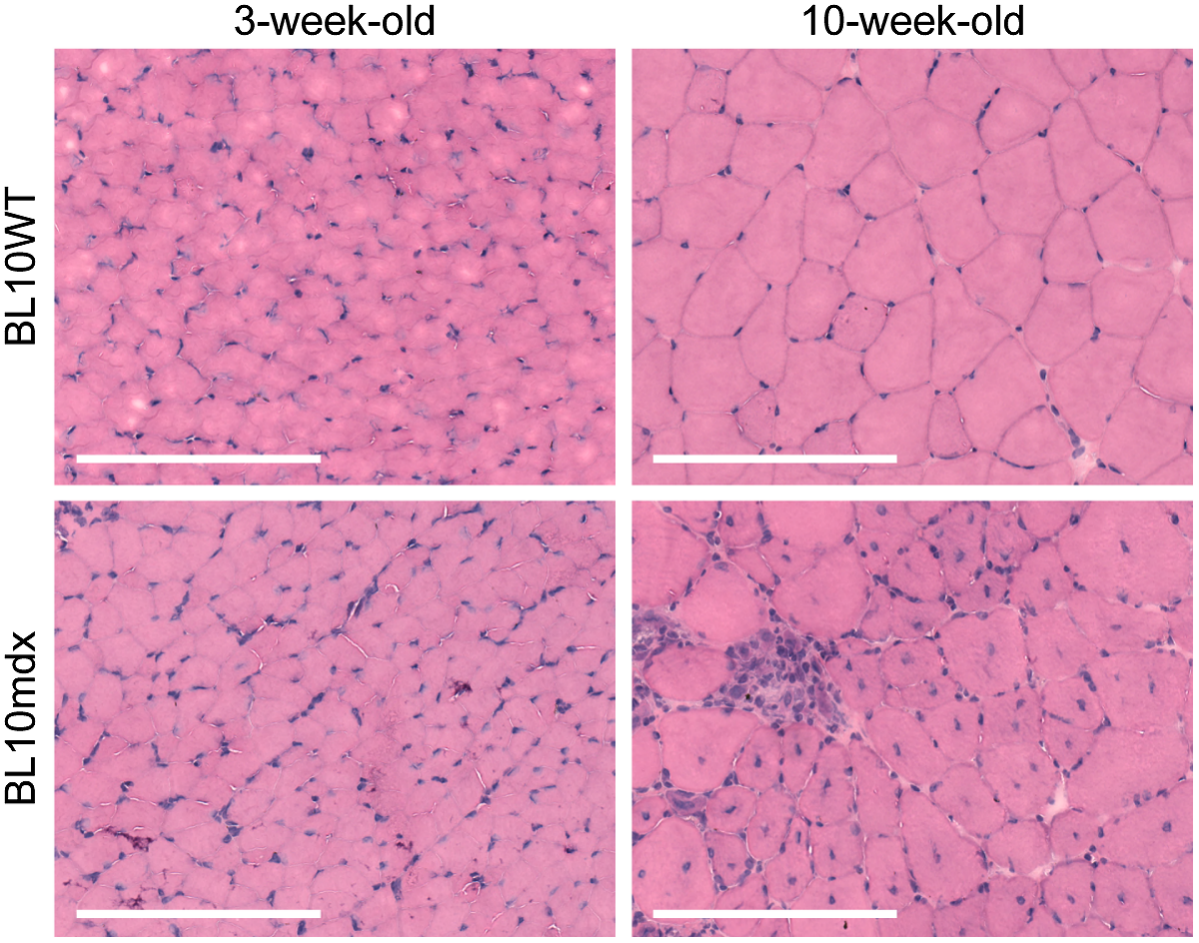
Representative histopathology of quadriceps muscle from 10-week-old *mdx* mice revealed several dystrophic features, including centralized nuclei and muscular atrophy by H&E not evident in 3-week-old *mdx* mice and aged B10 controls. Scale bars indicate 200  μm.

Histology of B10*mdx* mouse quadricep (Fig. 6) muscle found features of dystrophy consistent with the literature.^53^ This included centralized nuclei and degenerating/regenerating muscle fibers as seen using H&E histological staining. 3-week-old B10*mdx* mouse muscle showed no pathological features, comparable to B10WT controls.

## 4 Discussion

In this study, we describe the construction and testing of a fiber-optic AF/DR probe with significant potential for *in situ* analysis of biological tissues. The system relies on the native tissue optical properties in contrast to a growing series of studies that use custom-engineered exogenous markers including quantum dots,^5^ carbon nanotubes,^6^ and other nanomaterials,^8^ chiefly operating in the near-infrared wavelengths. At visible excitation wavelengths, AF and DR spectroscopies are more limited by tissue scattering in comparison with NIR. However, in applications such as skeletal muscle in which tissues are easily accessible, independence from exogenous dyes is advantageous.

UV light sources are typical in AF spectroscopy. Strong fluorophore absorption in this spectral region, predominantly by amino acids, ensures strong excitation efficiency and a broad fluorescence emission spectrum for analysis. However, given a monotonic decrease in tissue scattering coefficient with wavelength and a rapid drop in absorption coefficient across the visible spectrum (400 to 750 nm) in most tissues,^54^^,^^55^ deeper penetration of illumination light can be achieved using longer wavelengths.^56^^,^^57^ Visible laser sources are in addition safer, cheaper, and more efficiently guided by standard optical fiber in comparison with UV wavelengths. As such, a suitable balance between the variables of excitation efficiency, tissue penetration, cost, and safety were achieved in this study using 405 nm. At this wavelength, the exogenous fluorophores most efficiently excited include porphyrins, phospholipids, collagen, and flavins, such as FAD.^10^

Spectral examination of different skeletal muscles and myocardium showed a separability of these muscle types. This is consistent with the data of Chagnot et al.^24^ and Schilders and Gu^23^ who showed intrinsic AF differences between muscle fiber types using deep ultraviolet and visible wavelength excitation microscopy, respectively. A major source of variance between muscle types was observed between 500 and 600 nm for both AF and DR measurements, as is evidenced by principal components weightings in this region [Figs. 2(b) and 2(f)]. For DR measurements, this can be attributed to the combined absorption by Mb and Hb in both their oxygenated and deoxygenated states,^58^^,^^59^ whereas an additional absorption feature at ∼760  nm, evident only in cardiac muscle DR spectra [Fig. 3(e)], can be attributed to deoxygenated forms of both Mb and Hb.^60^^,^^61^ Deoxy-Mb and Hb exist in all muscle tissues, so this absorption may be preferentially elevated in cardiac muscle due to a higher concentration of these chromophores. Notably in these studies, we analyzed AF and DR data separately to compare the value of both modalities. Fiber-optic AF spectra are inherently a hybrid signal affected by AF and diffuse scattering and photon absorption, so integrating its analysis with DR would be questionable and certainly unconventional.

Two murine models of muscle disease that featured different phenotypes were investigated. The first was a model displaying limb-specific deletion of *Nf1*, which has been reported to develop intramyocellular lipid as well as weakness.^18^ The second model was the *mdx* mouse, which progressively develops a muscular dystrophy phenotype caused by structural deficiencies in the myofiber membrane.^53^ NF1 mice were separable from wild-type mice using DR and AF spectroscopy on the quadriceps, whereas measurements on cardiac muscle were inseparable [Figs. 3(d) and 3(h)]. This result is consistent with histological findings of increased intramyocellular lipid, likely having different optical properties to muscle without lipid droplets. Fibrotic tissues contain a higher density of collagen, making them more fluorescent in comparison with healthy muscle tissue in which AF in the visible region is derived by weaker concentrations of mitochondrial fluorophores, such as NADH.^10^ Differences in DR spectra for NF1 measurements in skeletal muscles could be caused by increased adiposity as adipose tissue is a weak absorber of visible light,^62^ resulting in brighter reflectance spectra and weaker fluorescence spectra. Importantly, and regardless of the underlying differences in optical properties, these muscle types were separable when approaching the spectral data with PCA-based dimensionality reduction and machine learning techniques.

The *mdx* mice again showed a separability based on both age and genotype, which was best observed using AF spectroscopy. This finding is consistent with those of Nakae et al.^26^ who attributed this phenomenon to the build-up of fluorescent lipofuscin granules in dystrophic murine and human samples. Notably, the B10 3-week and 10-week mouse quadriceps were not optically separable by AF/DR, whereas the *mdx* mice were. This indicates that the progressive dystrophic phenotype does shift the AF/DR spectra over time, which in a translational setting may enable disease progression to be followed longitudinally. An increase in lipofuscin accumulation has been demonstrated with age in both *mdx* and control mice, the latter of which only presents after 20 weeks.^26^ Importantly, from this time point, dystrophic muscle remains optically separable from controls due to a net higher concentration of the fluorophore throughout all time.

A limitation of this study was that the B10 controls were not littermate controls, and there may have been genetic drift with the *mdx* strain to confound a direct comparison. Furthermore, the use of recently euthanized mice rather than live mice and the biochemical and morphological differences between human and murine muscle tissue^63^ promote the need for further *in vivo* validation studies. Such studies may be supported by a more streamlined fiber-optic bundle with fewer fibers allowing long-term percutaneous access.

More robust methods of spectral calibration and analysis may also be beneficial. AF proved more diagnostically accurate than DR across all studies. When using fiber-optic geometries in optically turbid media, intrinsic fluorescence emissions are elastically scattered and absorbed as the photons traverse the tissue volume between sites of fluorophore emission and fiber-optic collection. As such, fiber-optic AF spectra are hybrid in nature and sensitive to the same optical processes that dominate DR. It is not obvious from our data whether this sensitivity enhances or degrades AF diagnostic efficacy, but different approaches to spectral analysis may prove more informative. It is worth noting that the PCA-based spectral feature extraction and noise rejection used in this study do not necessarily facilitate the understanding of the physical processes underpinning our results as complex PC weightings were difficult to assign biological significance. Other approaches that rely on detailed modeling of physical processes have enabled intrinsic AF extraction using a well-calibrated DR spectrum in combination with light transport models based on tissue phantom data, diffusion theory,^64^ Monte Carlo simulations,^65^ and other simpler numerical methods.^66^^,^^67^ Such an approach may also be complemented by the use of functional PCA^68^ instead of its more conventional form described in this article. If implemented in the context of this study, such methods may realize a more robust diagnostic system that could function across a more diverse range of hardware and sample populations in which factors such as tissue blood volume, probe geometry, contact pressures, and other hardware specifications are not as easily controlled for.

## 5 Conclusion

We describe the design of a minimally invasive marker-free method of optical spectroscopy suitable to the real-time *in vivo* analysis of muscle tissue. The system was implemented in healthy and diseased mouse tissues, including models of muscle myopathy seen with NF1-deficiency in mice and muscular dystrophy caused by *dystrophin* deficiency. Statistically transparent spectral analysis methods based on feature extraction by PCA and a QDC proved capable of distinguishing between healthy skeletal muscle types and diseased tissues. These data show the feasibility of using a fibre-optic AF and DR spectrometry system to characterize muscle tissue and underlying disease. This study serves as a strong proof-of-principle for further development of hardware and data analysis methods for clinical translation. To date these findings are largely empirical, and further study of autofluorescent macromolecules will be needed to appreciate the mechanisms underlying these spectral changes.
