# Supplementary material for: Analysis of muscle tissue in vivo using fiber-optic autofluorescence and diffuse reflectance spectroscopy
Source: J Biomed Opt. 2021 Dec 22;26(12):125001. doi: 10.1117/1.JBO.26.12.125001 (PMC8692235; doi:10.1117/1.JBO.26.12.125001)
Supplement: Supplementary file 1 [file JBO_026_125001_SD001.pdf]

Supplementary Material

1. Principal Component Feature Extraction, Dimensionality Reduction and Classifier Training

PCA is an unsupervised (blind to known observation classes) matrix transformation method that uses the covariance matrix of a dataset to construct an orthogonal basis of eigenvectors (features), known as principal components (PCs), each a linear combination of original variables and sorted by their eigenvalues such that each PC explains a consecutively diminishing percentage of the data's total variance. Geometrically projecting an original data point onto each PC assigns it a set of scores, one for each PC, which can be used for analysis and classification. The projection of a matrix of observations into a matrix of PC scores can be represented as $T = X_N W$, where X_N is a n×p matrix containing rows of spectra with n observations and p wavelengths, T is an n×q matrix of scores for n number of observation and q number of PCs (p>q), while W is a p×q matrix defining each of the q PC's weighting with respect to all p number of original variables (Fig. 1). A set of p minus q low-variance-explaining PCs can be rejected to remove noise and reduce the dataset's dimensionality by reducing the number of scores assigned to each original observation. and visualised to reveal the spectral shape of important features within the original data.

During a 5-fold cross validation process, a matrix of normalised spectra, X_N, was randomly permuted and divided into 5 equal folds. For the first round of training and validation, four folds were combined as training data, $X_{N,train}$, while one fold was held out for testing, $X_{N,test}$. Training data underwent PCA transformation, generating a set of high variance-explaining PCs, the total number of which was decided after optimisation to be three. This matrix of PCs, W_{train}, was used to transform $X_{N,train}$ into a scores matrix, T_{train}, which, along with corresponding class labels, y_{train}, was used to train a QDC. W_{train} was then used to transform $X_{N,test}$ into a matrix of scores, T_{test}, which were then classified by the trained QDC model. This process was repeated using all fold permutations, each training a new QDC and cumulatively collecting validation predictions into a confusion matrix allowing the calculation of averaged performance metrics. This method ensured that the QDC was exposed to the whole dataset while remaining blind to the test data at all times.

Trained classification models base each classification event off a calculated probability that a data point belongs to the positive class by applying a threshold below which it assigns a

classification of false and above which it assigns a classification of true [34]. Changing the probability threshold affects classification performance with respect to true positive rate (TPR) and false positive rate (FPR). In any given application of the model, a user's concern for sensitivity (TPR) and specificity (1-FPR) may vary. Therefore, by considering both TPR and FPR over a range of possible QDC probability thresholds, a more general performance metric is assigned to each model than would be described using other more precise metrics such as accuracy, recall or f-score. An ROC curve for each trained and tested model was therefore plotted by combining the true positive rate (TPR) and false positive rate (FPR) for a range of different classification probability thresholds. An ROC area under the curve (AUC) near 1 indicates a strong classifier, 0.5 indicates a weak classifier and 0 indicates a perfect inverse classifier.

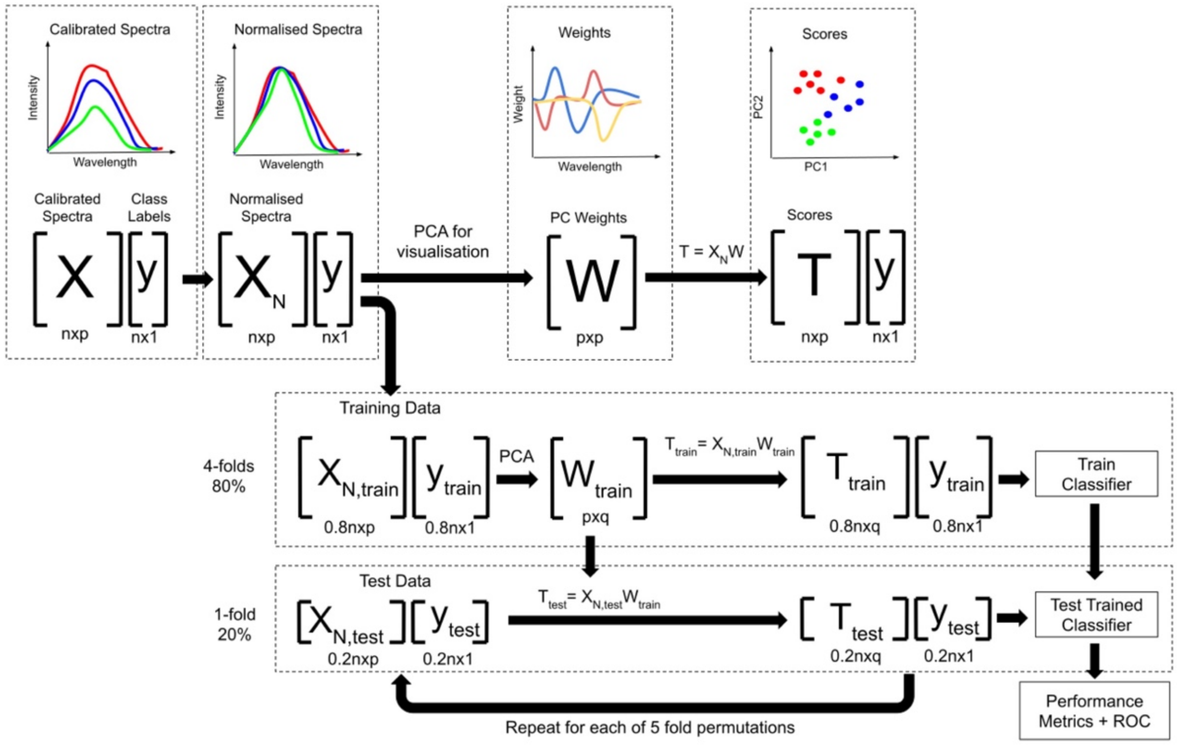

Fig. 1: Spectral Pre-processing, Data Visualisation and Classification Model Training.

2. Pre-processing and Dimensionality Reduction Optimisation

It was postulated that the normalisation of calibrated spectral data and subsequent dimensionality reduction with PCA would enhance classification performance using a QDC. The extent of dimensionality reduction first had to be optimised to enable sufficient reduction in dataset size to expose important sources of variance, while not hindering the ability of the QDC to separate muscle classes. As per the methods outlined in Supplementary Methods 1, data from BL6 mice, including measurements from all 3 skeletal muscle types and cardiac muscle, were grouped into a single matrix. PCA dimensionality reduction was performed directly on this matrix, and separately on a normalised form of the same matrix. Scores matrices were constructed using a different number of PCs (1-10). Results from QDC classification of each of these matrices were summarised using the area under the curve of each classifier's receiver operator curve (Fig. 2A,D).

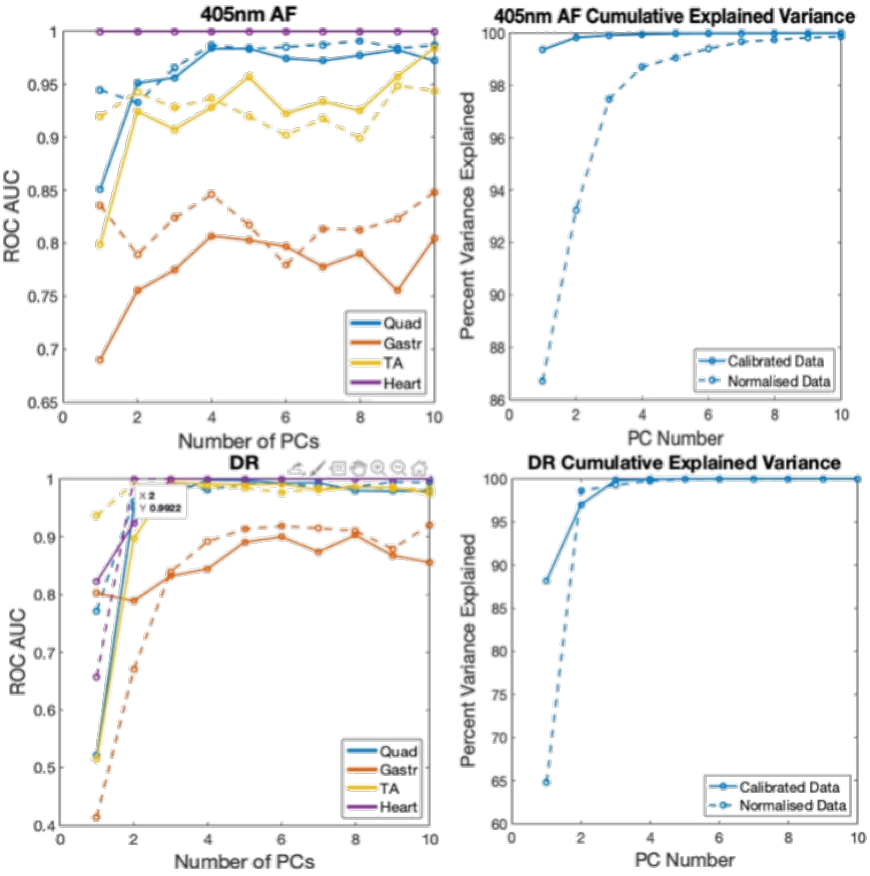

Fig. 2. **Left column**: Classification performance of PCA-based models as measured using receiver operator area-under-the-curve plateaued using 3 PCs for both 405nm AF and DR. Solid lines show results for calibrated (not normalised) data, while dotted lines show that for normalised data. **Right column**: For datasets including results for all muscle types, more than 95% of variance was explained by the first 3 PCs. These findings suggest that using 3 PCs in an AF/DR predictive model provides a suitable compromise between dimensionality reduction and capture of inherent variance.

Both AF and DR classification performance were found to benefit from spectral normalisation when using less than 5 PCs, and similar performances were observed between normalised and original spectral datasets for more than 5 PCs (Fig. 2A,C).

The first 3 principal components extracted from this combined matrix of BL6 muscle types accounted for more than 97% of total variance in both AF and DR normalised and original datasets (Fig. 2 B,D). Classification performance was seen to plateau upon the addition of more than 3 PCs to the model for most muscle types, making the inclusion of more PCs redundant, exposing the model to noise.
